# Serologic evidence of pandemic (H1N1) 2009 virus infection in camel and Eld’s deer, Thailand

**DOI:** 10.14202/vetworld.2021.2596-2601

**Published:** 2021-10-05

**Authors:** Somjit Chaiwattanarungruengpaisan, Natthaphat Ketchim, Wanvisa Surarith, Metawee Thongdee, Phirom Prompiram, Kanittha Tonchiangsai, Wanlaya Tipkantha, Witthawat Wiriyarat, Weena Paungpin

**Affiliations:** 1 The Monitoring and Surveillance Centre for Zoonotic Diseases in Wildlife and Exotic Animals, Faculty of Veterinary Science, Mahidol University, Nakhon Pathom, Thailand; 2 The Zoological Park Organization of Thailand, Bureau of Conservation and Research, Bangkok, Thailand.

**Keywords:** camel, Eld’s deer, pandemic (H1N1) 2009 virus, serosurveillance, Thailand

## Abstract

**Background and Aim:**

The pandemic (H1N1) 2009 influenza (H1N1pdm09) virus has affected both human and animal populations worldwide. The transmission of the H1N1pdm09 virus from humans to animals is increasingly more evident. Captive animals, particularly zoo animals, are at risk of H1N1pdm09 virus infection through close contact with humans. Evidence of exposure to the H1N1pdm09 virus has been reported in several species of animals in captivity. However, there is limited information on the H1N1pdm09 virus infection and circulation in captive animals. To extend the body of knowledge on exposure to the H1N1pdm09 virus among captive animals in Thailand, our study investigated the presence of antibodies against the H1N1pdm09 virus in two captive animals: Camelids and Eld’s deer.

**Materials and Methods:**

We investigated H1N1pdm09 virus infection among four domestic camelid species and wild Eld’s deer that were kept in different zoos in Thailand. In total, 72 archival serum samples from camelid species and Eld’s deer collected between 2013 and 2014 in seven provinces in Thailand were analyzed for influenza antibodies using hemagglutination inhibition (HI), microneutralization, and western blotting (WB) assays.

**Results:**

The presence of antibodies against the H1N1pdm09 virus was detected in 2.4% (1/42) of dromedary camel serum samples and 15.4% (2/13) of Eld’s deer serum samples. No antibodies were detected in the rest of the serum samples derived from other investigated camelids, including Bactrian camels (0/3), alpacas (0/5), and llamas (0/9). The three positive serum samples showed HI antibody titers of 80, whereas the neutralization titers were in the range of 320-640. Antibodies specific to HA and NP proteins in the H1N1pdm09 virus were detected in positive camel serum samples using WB. Conversely, the presence of the specific antibodies in the positive Eld’s deer serum samples could not be determined using WB due to the lack of commercially labeled secondary antibodies.

**Conclusion:**

The present study provided evidence of H1N1pdm09 virus infection in the captive dromedary camel and Eld’s deer in Thailand. Our findings highlight the need for continuous surveillance for influenza A virus in the population of dromedary camels and Eld’s deer. The susceptible animal populations in close contact with humans should be closely monitored. Further study is warranted to determine whether Eld’s deer are indeed a competent reservoir for human influenza virus.

## Introduction

Over a decade after its emergence in 2009, the pandemic (H1N1) 2009 virus (H1N1pdm09 virus) has spread worldwide and continues to circulate as a seasonal influenza virus [1]. The H1N1pdm09 virus has affected not only the human population but also the animal populations. Evidence of virus infection has been found in diverse mammalian species, including American badger, Bornean binturong, Northern elephant seal, black-footed ferret, pet ferret, cheetah, cat, camel, elephant, guinea pig, dog, giant panda, pig, tiger, turkey, and skunk [2,3]. Virus spillover from humans to susceptible animal species may result in an alternative virus reservoir [3]. Moreover, host switching may contribute to viral genetic changes (evolution, mutation, and gene reassortment) that lead to the emergence of a new virus [3,4]. Hence, the identification of susceptible animals and transmission within the animal populations is important for the assessment of zoonotic potential.

Sufficient interaction between infected humans and recipient animals is required for cross-species transmission [3]. At present, a large number of animals are maintained in captivity for various purposes, including farming, conservation, education, and tourism. These captive animals, especially zoo animals, are considered to be in frequent contact with humans. Close contact between humans and animals may facilitate virus transmission from humans to animals and vice versa. The previous studies have reported the infection of the H1N1pdm09 virus in several captive animals, such as American badger, Bornean binturong, black-footed ferret, cheetah, and giant panda [5-8]. In Thailand, there was evidence of H1N1pdm09 virus exposure in Asian elephants [9] and tigers [10]. However, the serological and epidemiological studies in captive animals are still limited.

To extend the body of knowledge surrounding H1N1pdm09 virus exposure in captive animals in Thailand, we investigated the presence of antibodies against the H1N1pdm09 virus in two captive animals: camelids and Eld’s deer. The infection of H1N1pdm09 virus has not previously been determined in the populations of both animal species in Thailand. Serological evaluations, comprising the hemagglutination inhibition (HI), microneutralization (MN), and western blotting (WB) assays, were performed for antibody detection.

## Materials and Methods

### Ethical approval

This study was approved by Faculty of Veterinary Science, Mahidol University-Institute Animal Care and Use Committee (No. MUVS-2018-01-04).

### Study period and location

Blood samples were collected from camelid species and Eld’s deer during the Year 2013 -2014. Those captive animals live in zoos located in seven provinces across the country, including Chiang Mai (Northern), Khon Kaen and Nakhon Ratchasima (Northeastern), Chon Buri (Eastern), Bangkok and Suphan Buri (Central), and Ratchaburi (Western).

### Archival sera

**Figure-1:**
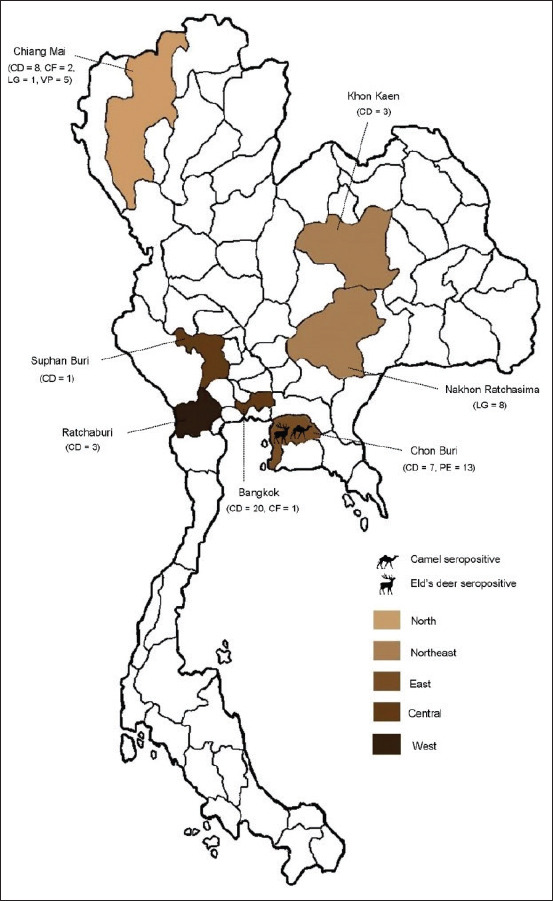
Map of Thailand depicting locations at which camelid species and Eld’s deer were collected. A number of samples derived from each animal species are indicated in the parentheses. Abbreviation: CD; *Camelus dromedarius*, CF; *Camelus ferus*, LG; *Lama glama*, PE; *Panolia eldii*, VP; *Vicugna pacos* [Source: https://commons.wikimedia.org/wiki/File: BlankMap_Thailand.png.}

In total, 72 archival serum samples of camelid species and Eld’s deer were used in the study. The serum samples were obtained from four domestic camelids, including alpaca (n=5), Bactrian camel (n=3), dromedary camel (n=42), and llama (n=9); and wild Eld’s deer (n=13). All animals were kept in zoos. The camelid serum samples were collected under a survey project for Middle East respiratory syndrome and other important diseases in camelids in Thailand [11]. Eld’s deer serum samples were obtained from the disease surveillance program conducted through the collaboration between the Monitoring and Surveillance Center for Zoonotic Diseases in Wildlife and Exotic Animals (MoZWE), Faculty of Veterinary Science, Mahidol University, and the Zoological Park Organization of Thailand. Most of the animals had no respiratory diseases or clinical symptoms at the time of blood collection. The blood samples were analyzed in the MoZWE laboratory. The serum was separated and stored at −80°C until analysis. The numbers and habitat locations of each animal are shown in Figure-1 and Table-1.

**Table-1:** Demographic characteristics of archival sera derived from four camelid species and Eld’s deer living in different zoos in Thailand.

Species	Common name	Year	Province	Region	Number
*Camelus dromedarius*	Dromedary camel	2014	Bangkok	Central	20
			Chiang Mai	Northern	8
			Chon Buri	Eastern	7
			Khon Kaen	Northeastern	3
			Ratchaburi	Eastern	3
			Suphan Buri	Central	1
*Camelus ferus*	Bactrian camel	2014	Bangkok	Central	1
			Chiang Mai	Northern	2
*Lama glama*	Llama	2014	Chiang Mai	Northern	1
			Nakhon Ratchasima	Northeastern	8
*Panolia eldii*	Eld’s deer	2013	Chon Buri	Eastern	13
*Vicugna pacos*	Alpaca	2014	Chiang Mai	Northern	5
Total					72

### The study viruses

The H1N1pdm09 virus, A/Thailand/104/2009(H1N1), was kindly provided by Professor Emeritus Dr. Pilaipan Puthavathana. The complete genome sequencing of this virus can be retrieved from GenBank, with accession number GQ169382 for the HA nucleotide sequence and accession number ACR23302 for the HA amino acid sequence. The H1N1pdm09 virus was propagated in Madin–Darby canine kidney (MDCK) cells (American Type Culture Collection: CCL-34) maintained in viral growth media containing Eagle’s minimum essential medium (Gibco, Grand Island, USA) supplemented with 2 μg/mL of trypsin-tosyl phenylalanyl chloromethyl ketone (trypsin-TPCK) (Sigma-Aldrich, St. Louis, USA). The propagation of the virus was performed at the Biological Safety Levels-2 facility of the Faculty of Veterinary Science, Mahidol University.

### HI assay

The HI assay was performed as a screening test for the detection of antibodies against the pandemic human H1 subtype. All archival serum samples were treated with receptor-destroying enzyme (Denka Seiken Co. Ltd., Tokyo, Japan) at 37°C for 16 h, followed by heat inactivation at 56°C for 30 min, and absorption with 50% goose erythrocyte suspension at 4°C for 1 h. The HI assay was performed in accordance with a previously described procedure [12]. The serum samples with an HI titer of ≥20 were further analyzed for neutralizing antibodies using the MN assay.

### MN assay

The MN assay was conducted as previously described [9,12]. Briefly, the treated serum was mixed with the test virus (final concentration of 100 TCID50/well) and incubated at 37°C for 2 h. The serum-virus mixture was transferred onto a monolayer of MDCK cells and then further incubated at 37°C for 2 days. The cell monolayers were examined for the presence of a cytopathic effect, whereas the culture supernatants were analyzed for non-neutralized viruses using the hemagglutination assay. A neutralization titer (NT) of ≥20 was considered as a positive result. The HI- and NT-positive serum samples were analyzed to confirm the presence of influenza A virus infection by WB analysis.

### WB assay

The WB assay was performed in accordance with the published protocol [9]. The individual test antigen was mixed with reducing sample buffer (8% sodium dodecyl sulfate [SDS], 250 mM Tris-HCl pH 6.8, 8% β-mercaptoethanol, 0.4% bromophenol blue, and 40% glycerol) and boiled for 10 min before separation using 12% SDS-PAGE. The proteins resolved on the gel were then blotted onto a nitrocellulose membrane (Bio-Rad, Hercules, USA) using a TE77X semidry transfer unit (Hoefer Inc., Holliston, USA). Non-specific binding to the membrane was blocked by incubation in 1% bovine serum albumin in PBS plus 0.1% Tween-20. The positive and negative sera were diluted 1 in 50, and the secondary antibody was diluted 1 in 5,000. Finally, a DAB (3,3′-diaminobenzidine tetrahydrochloride) substrate kit (Thermo Scientific, Waltham, USA) was used as the chromogenic substrate.

## Results

### HI assay for antibody to influenza A virus

The serum samples were initially screened for the presence of antibodies against the pandemic human H1 subtype using the HI assay. Of the 72 serum samples tested, 3 (1.4%) samples were positive for human H1 virus. The overall geometric mean titers of antibodies against the human H1 virus in camelid species and Eld’s deer were 10.9. The HI-positive serum samples were obtained from one dromedary camel and two Eld’s deer, indicating that the positivity rate among these species was 2.4% (1/42) and 15.4% (2/13), respectively. All three positive serum samples had a HI antibody titer of 80 against human H1 virus.

### MN assay for antibodies to the H1N1pdm09 virus

Further confirmatory testing by the MN assay was performed on the HI-positive serum samples. The inhibition of H1N1pdm09 virus infection in MDCK cells was observed in all three positive serum samples. The NT antibody titer of the HI-positive serum samples of the dromedary camel and two Eld’s deer was defined at dilutions of 1:640, 1:320, and 1:640, respectively. The HI/NT titer was determined for each positive serum sample. The positive dromedary camel serum with ID 1560/14 had an HI/NT titer of 80/640, whereas the two positive Eld’s deer with IDs of 671/13 and 674/13 had HI/NT titers of 80/320 and 80/640, respectively.

### WB assay for antibody to H1N1pdm09 virus

**Figure-2:**
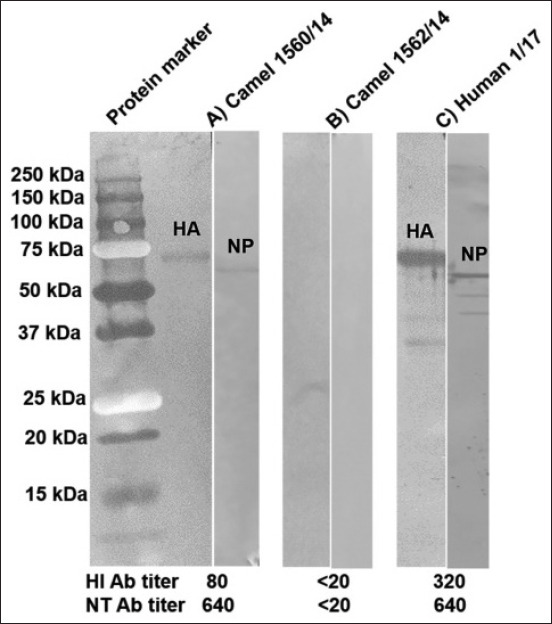
WB assay for the detection of antibody to HA and NP proteins of H1N1pdm09 virus in the camel serum. Recombinant proteins of HA and NP were used as the test antigen and rabbit anti-camel conjugate-HRP was used as the secondary antibody. The rHA (MW=59 kDa) and rNP (MW=53.5 kDa) proteins were detected by antibodies in the camel serum sample ID 1560/14 with HI antibody titer of 80 (A) as well as the convalescent serum from an H1N1pdm09 patient (C) but neither HA nor NP protein was detected by the camel serum sample ID 1562/14 with HI antibody titer <20 (B).

Human H1 influenza virus infection in the dromedary camel was confirmed by WB analysis using rabbit anti-camel conjugate-HRP (Mybiosource.com, San Diego, USA) as the secondary antibody. The recombinant HA (rHA) and recombinant NP (rNP) proteins of the H1N1pdm09 virus purchased from Sino Biological Inc., China (catalog numbers: 11055-V08B and 40205-V08B, respectively), were used as the test antigen. The results of the WB assay demonstrated that the positive dromedary camel serum (ID 1560/14; HI/NT titer of 80/640) recognized the rHA and rNP proteins with a molecular weight of 59 and 53.5 kDa, respectively(Figure-2). A convalescent serum sample from an H1N1pdm09-infected volunteer (ID: human 1/17; HI/NT titer of 320/640) was also recognized by the same band of the rHA and rNP proteins (Figure-2). Conversely, there were no antibodies in the negative dromedary camel serum (ID: 1562/14 serum; HI/NT titer of <20) that recognized the rHA and rNP proteins of the H1N1pdm09 virus (Figure-2). The results of the WB assay clearly demonstrated the presence of anti-HA and anti-NP antibodies of the H1N1pdm09 virus in the positive camel serum. In the positive serum samples of two Eld’s deer, confirmation could not be performed by WB due to the lack of commercially labeled secondary antibodies.

## Discussion

In this study, we investigated exposure to the H1N1pdm09 virus among in camelid species and wild Eld’s deer that were kept in zoos in Thailand. Camelid species were imported to Thailand more than a decade ago. Four camelid species found in Thailand are composed of two species of old-world camelids (Bactrian camel and dromedary camel) and two species of new-world camelids (alpaca and llama). The imported camelids have been kept in captivity in several parts of the country, notably in zoos, farms, and private resorts. Up to 70 camelids were counted during 2014, the period when the camelid serum used in this study was collected (personal communication). In recent years, an increasing proportion of camelids in Thailand have been associated with tourism. In contrast, Eld’s deer is considered as an endangered species of deer. This threatened species has been on the International Union for the Conservation of Nature’s Red List since 2008 [13]. Approximately 100 Eld’s deer are estimated left in Thailand [14]. Half of those animals were bred and raised at the breeding center.

The presence of antibodies against the H1N1pdm09 virus was detected in 2.4% (1/42) of dromedary camel serum samples and 15.4% (2/13) of Eld’s deer serum samples. Antibodies were not detected in the rest of the serum samples derived from other investigated camelids, including Bactrian camel (0/3), alpaca (0/5), and llama (0/9). The three positive serum samples had HI antibody titers of 80, whereas the NT antibody titers were in the range of 320–640. WB analysis was used to confirm the HI- and NT-positive results. Antibodies specific to the HA and NP proteins of the H1N1pdm09 virus were successfully detected in the positive camel serum sample by WB. Conversely, the presence of those specific antibodies in the positive Eld’s deer serum samples could not be confirmed by WB due to lack of commercially labeled secondary antibodies. However, this is the first report providing evidence of H1N1pdm09 virus infection in the captive dromedary camel and Eld’s deer in Thailand.

Several studies have reported influenza virus infection in camelid species. In the past, a reassortant influenza A (H1N1) virus vaccine strain, possibly transmitted from vaccinated humans, was shown to be responsible for fatal epizootics among Bactrian camels in Mongolia [15]. Serologic activity has been demonstrated against influenza A virus among camels in Sudan [16] and against influenza A and B viruses in Nigeria [17]. Seropositivity to H1-like influenza A virus was found in llamas in Argentina [18]. Recently, the partial genome of influenza A virus was detected in the imported dromedary camels from Sudan and Djibouti between 2017 and 2018 in Saudi Arabia. The analysis of this partial genome sequence of the influenza A virus revealed a close relationship to human/swine influenza A (H1N1) isolates from 2009 to 2019 in various countries [19]. In addition, a recent study demonstrated influenza A virus infection in dromedary camels in Nigeria and Ethiopia [20]. Antibodies against the H1N1 influenza virus were found in four serum samples of dromedary camels in Ethiopia by HI assay, whereas antibodies against H3N2 influenza virus were also found in one serum sample from a dromedary camel in Nigeria in the MN assay. Unexpectedly, the H1N1pdm09-like virus was first isolated from a nasal swab of a dromedary camel in Nigeria. The whole genome of camel influenza virus has been sequenced. Eight genome sequences (GenBank accession no. MN453859-66) showed highest identity (>99.8) with contemporary H1N1pdm09 viruses [20]. Although our study did not detect the influenza virus genome in the nasal swab samples acquired from some of the investigated dromedary camels (data not shown), we were able to demonstrate the seropositivity to H1N1pdm09 virus in a dromedary camel using the HI, MN, and WB assays. In contrast, influenza virus infection has never been reported in Eld’s deer. In our study, antibodies to the H1N1pdm09 virus were detected in two serum samples of wild Eld’s deer using the HI and MN assays, indicating past infection of the virus in those captive species. Further investigation of influenza virus exposure in camelid species and Eld’s deer should include the currently circulating strains of both human and avian influenza viruses in the serology assays.

The presence of H1N1pdm09 virus antibody in both dromedary camel and Eld’s deer implied the susceptibility of those animal species to natural infection. The cross-species transmission of human influenza virus to dromedary camel has been previously demonstrated, whereas the transmission of human influenza virus to Eld’s deer requires further elucidation. Seropositivity to the H1N1pdm09 virus was found in Eld’s deer serum samples collected in 2013 and a dromedary camel serum sample collected in 2014. Those seropositive animals were housed in two different open zoos located in Chon Buri, a well-known seaside province of Thailand. From the available data, the seropositive animals were adult females, and the seropositive dromedary camel was 10 years old and pregnant at the time of blood collection. The source of H1N1pdm09 virus infection is unknown. However, it is most likely acquired from animal caretakers or tourists who participated in zoo activities, such as animal breeding and feeding.

## Conclusion

The past infection of H1N1pdm09 virus was first determined in a captive domestic dromedary camel and wild Eld’s deer in Thailand. Our findings highlight the need for continuous surveillance for influenza A virus in dromedary camels and Eld’s deer, especially in animal populations that come into close contact with humans. Further study is warranted to determine whether Eld’s deer is indeed a competent reservoir for the human influenza virus.

## Authors’ Contributions

SC, NK, WS, MT, and PP: Performed laboratory tests. NK, KT, WT, and WW: Sample collection. WP: Designed the experiment and analyzed the data. WP and MT: Drafted and revised the manuscript. All authors read and approved the final manuscript.
